# Mast Cell Activation in Brain Injury, Stress, and Post-traumatic Stress Disorder and Alzheimer's Disease Pathogenesis

**DOI:** 10.3389/fnins.2017.00703

**Published:** 2017-12-12

**Authors:** Duraisamy Kempuraj, Govindhasamy P. Selvakumar, Ramasamy Thangavel, Mohammad E. Ahmed, Smita Zaheer, Sudhanshu P. Raikwar, Shankar S. Iyer, Sachin M. Bhagavan, Swathi Beladakere-Ramaswamy, Asgar Zaheer

**Affiliations:** ^1^Department of Neurology and Center for Translational Neuroscience, School of Medicine, University of Missouri, Columbia, MO, United States; ^2^Harry S. Truman Memorial Veteran's Hospital, United States Department of Veterans Affairs, Columbia, MO, United States

**Keywords:** Alzheimer's disease, mast cells, neurodegeneration, neuroinflammation, stress, PTSD

## Abstract

Mast cells are localized throughout the body and mediate allergic, immune, and inflammatory reactions. They are heterogeneous, tissue-resident, long-lived, and granulated cells. Mast cells increase their numbers in specific site in the body by proliferation, increased recruitment, increased survival, and increased rate of maturation from its progenitors. Mast cells are implicated in brain injuries, neuropsychiatric disorders, stress, neuroinflammation, and neurodegeneration. Brain mast cells are the first responders before microglia in the brain injuries since mast cells can release prestored mediators. Mast cells also can detect amyloid plaque formation during Alzheimer's disease (AD) pathogenesis. Stress conditions activate mast cells to release prestored and newly synthesized inflammatory mediators and induce increased blood-brain barrier permeability, recruitment of immune and inflammatory cells into the brain and neuroinflammation. Stress induces the release of corticotropin-releasing hormone (CRH) from paraventricular nucleus of hypothalamus and mast cells. CRH activates glial cells and mast cells through CRH receptors and releases neuroinflammatory mediators. Stress also increases proinflammatory mediator release in the peripheral systems that can induce and augment neuroinflammation. Post-traumatic stress disorder (PTSD) is a traumatic-chronic stress related mental dysfunction. Currently there is no specific therapy to treat PTSD since its disease mechanisms are not yet clearly understood. Moreover, recent reports indicate that PTSD could induce and augment neuroinflammation and neurodegeneration in the pathogenesis of neurodegenerative diseases. Mast cells play a crucial role in the peripheral inflammation as well as in neuroinflammation due to brain injuries, stress, depression, and PTSD. Therefore, mast cells activation in brain injury, stress, and PTSD may accelerate the pathogenesis of neuroinflammatory and neurodegenerative diseases including AD. This review focusses on how mast cells in brain injuries, stress, and PTSD may promote the pathogenesis of AD. We suggest that inhibition of mast cells activation and brain cells associated inflammatory pathways in the brain injuries, stress, and PTSD can be explored as a new therapeutic target to delay or prevent the pathogenesis and severity of AD.

## Introduction

Mast cells derived from hematopoietic progenitors are multifunctional antigen presenting cells present in the tissues throughout the body (Kalesnikoff and Galli, 2008; Krystel-Whittemore et al., 2015; Galli and Gaudenzio, 2017). Mast cells are involved in both health and disease conditions by releasing specific inflammatory, anti-inflammatory, and other mediators in tissues (Gurish and Austen, 2001). Mast cells are involved in immune responses, inflammation, tissue damage, and repair mechanism of the damaged tissues in peripheral organs and in the central nervous system (CNS) (Amor and Woodroofe, 2014; Skaper, 2016). Human mast cells are broadly classified into mucosal type (MC_T_) and connective tissue type (MC_TC_), based upon the type of proteases present in their cytoplasmic granules. The mucosal type of mast cells contain tryptase while the connective tissue type of mast cells contain both tryptase as well as chymase in their cytoplasmic granules (Galli, 1990; Irani and Schwartz, 1994). Brain mast cells also show similar heterogeneity (Maslinska et al., 2007). Mouse primary mast cells express various mouse mast cell proteases (MMCPs) that are different from human mast cell proteases and are classified as connective tissue mast cells (CTMC) or mucosal type mast cells (MMC) based on the expression of MMCPs (Wernersson and Pejler, 2014).

Mast cells play an important role in inflammatory pathogenesis such as anaphylactic reactions, asthma, allergy, arthritis, cardiovascular diseases, systemic mastocytosis, interstitial cystitis, psoriasis, atopic dermatitis, cancer and metastasis, endometriosis, obesity, ulcers, prostatitis, periodontitis, irritable bowel syndrome (IBS), and inflammatory bowel disease (IBD) (Sant et al., 2007; Theoharides et al., 2008, 2010, 2012a; Galli and Tsai, 2012; Kritikou et al., 2016; Suurmond et al., 2016). Additionally, we and others have shown that mast cells are also implicated in many neurological and neuroinflammatory conditions including brain injury, traumatic brain injury (TBI), stroke, Multiple sclerosis (MS), Experimental Autoimmune Encephalomyelitis (EAE), Parkinson's disease (PD), dementia, Alzheimer's disease (AD), intracerebral hemorrhage (ICH), neuropsychiatric disorders, stress conditions, sleep disorders, migraine, pain, headache, attention deficit disorder, autism, joint and muscle pain, and itching (Theoharides et al., 1995, 2005, 2016; Sayed et al., 2011; Karagkouni et al., 2013; Graziottin et al., 2014; Kempuraj et al., 2016a,b; Moretti et al., 2016; Shaik-Dasthagirisaheb and Conti, 2016). Mast cell-derived inflammatory mediators increase blood brain barrier (BBB) permeability and activate brain resident immune cells such as microglia (Ribatti, 2015). Further, mast cell mediators increase vascular permeability and increase escape and recruitment of immune and inflammatory cells at the site of injury. Optimal inflammatory responses or physiological levels of inflammatory mediators are beneficial and protect the body as they remove unwanted waste materials and repair the damaged tissues. However, excessive and chronic inflammatory responses can lead to increased inflammatory mediator levels, severe inflammation and tissue injury. Mast cells grow in numbers by increased proliferation, increased recruitment, increased survival, and accelerated maturation from their progenitors during inflammatory conditions (Bulfone-Paus et al., 2017). Mast cells are ubiquitous in the body, but they are highly concentrated in the regions where the body is directly exposed to the outer environment, and also in inflammatory and allergic tissues (Wernersson and Pejler, 2014). Thus, mast cells participate in the first line of defense from the invading pathogenic organisms and other environmental factors including pollutants (Tsai et al., 2011; Wernersson and Pejler, 2014). Further, mast cells are one of the fastest responders by releasing prestored and newly synthesized mediators among immune and inflammatory cells (Bulfone-Paus et al., 2017). Mast cells are reported as the cells that are ready to battle any time with any kind of threats to the body, since their cytoplasmic secretory granules are filled with several preformed as well as preactivated immune and inflammatory mediators including histamine, tryptase, chymase, tumor necrosis factor-alpha (TNF-α), serotonin, heparin, proteoglycans, and vascular endothelial growth factor (VEGF) (Beil et al., 1994; Wernersson and Pejler, 2014; Yehya and Torbey, 2017). Mast cells can generate and release reactive oxygen species (ROS) within seconds of its activation (Yehya and Torbey, 2017). Mast cells are powerful and rapid sensors of tissue injury/necrosis, infectious agents, and inflammation since they are located in the host-environment boundaries and express pattern recognition receptors and cytokines such as local alarmin, interleukin-33 (IL-33) (Martin and Martin, 2016). Mast cells detect and rapidly respond to protect from cellular injuries by detecting IL-33 released from the damaged cells (Lunderius-Andersson et al., 2012). Mast cells not only sense the cellular microenvironment in the tissues but also sense and respond to external environment such as cold, hot, humidity, pressure, allergen, and toxins (Krause et al., 2010; Engebretsen et al., 2016; Wang et al., 2016).

Mast cells are highly armed defensive system similar to our soldiers and police, protecting the body from outside and inside threats, including invasion of infectious agents, parasites, injuries, and toxins. Mast cells perform these functions rapidly by releasing prestored mediators from their granules and by synthesizing and secreting specific new chemical substances/mediators as required in acute and chronic immune responses. Mast cell committed hematopoietic progenitors from bone marrow enters into the bloodstream, gets transported into the tissues/organs, settles there and matures into specific type of mast cells depending upon the type of tissue and microenvironment, with local specific growth factors and the specific needs/threat of that particular tissue. For example, the mast cells present in the skin are connective tissue type, and mast cells present in the lung are mucosal type. Connective tissue type of mast cells can respond extensively to neuropeptides such as substance P and neurotensin. However, mast cells are interchangeable and thus can transform into another phenotype whenever needed in the body. Moreover, mast cells travel to another region or tissue, settle there and sense the new tissue microenvironment, and then proliferate to increase specific mast cell type based upon the requirement. Factors like infections locally and transiently increases the accumulation of mast cell progenitors and increases mast cell numbers (Zarnegar et al., 2017). Mast cells even migrate in to the brain from peripheral organs through BBB and proliferate in the brain during neuroinflammatory conditions.

Mast cell activation leads to the release of specific inflammatory mediators such as TNF-α, IL-1β, IL-8, IL-33, chemokine (C-C motif) ligand 2 (CCL2), CCL3, CCL5, granulocyte macrophage colony-stimulating factor (GM-CSF), VEGF, matrix metalloproteinase (MMPs), ROS, substance P (SP), stem cell factor (SCF), nerve growth factor (NGF), dopamine, transforming growth factor-beta (TGF-β), corticotrophin-releasing hormone (CRH), neurotensin, histamine, prostaglandin D2 (PGD2), leukotrienes (LTs), proteases tryptase and chymase based upon the type of mast cells and type of stimuli in the tissues (Gilfillan et al., 2011; Wernersson and Pejler, 2014; Gaudenzio et al., 2016; Kempuraj et al., 2016b; Borriello et al., 2017). Mast cells express receptors and ligands for various immune and inflammation related pathways including CRH, SP, CD40, CD40L, SCF, cytokines, and chemokines (Kim et al., 2011; Theoharides, 2017). About 25% of rat mast cell granule content is TNF-α that is neurotoxic (Hendriksen et al., 2017). Inflammatory pathways in the brain with many of the above-mentioned mediators lead to neuroinflammation, which is an important process in the onset and progression of AD, PD, and MS (Zaheer et al., 2008; Chen et al., 2016; Kempuraj et al., 2016b). C-reactive protein (CRP), a marker of inflammation is implicated in mood disorders (De Berardis et al., 2006), cognitive disorders and AD (Finch and Morgan, 2007; O'bryant et al., 2016), and Post-Traumatic Stress Disorder (PTSD) (Solomon et al., 2017). A recent study showed increased blood levels of inflammatory markers IL-1β, TNF-α, IL-6, and IL-10 in Lewy body dementia (LBD) and increased CRP in PD dementia patients (King and Thomas, 2017). Another recent review reported possible peripheral inflammatory markers in AD patients (Lai et al., 2017). This report combined the results from 175 studies, analyzed 51 inflammatory markers in AD patients, and compared with control subjects. They reported increased peripheral levels of TNF-α converting enzyme, IL-1β, IL-2, IL-6, IL-18, interferon-gamma (IFN-γ), homocysteine, high-sensitivity CRP, CCL10, epidermal growth factor (EGF), vascular cell adhesion molecule-1 (VACM-1), TNF receptor1/2, and α1-antichymotrypsin in AD patients as compared with the levels in the control subjects (Lai et al., 2017). This study suggests that AD pathogenesis is associated with peripheral immune and inflammatory responses, and increased IL-6 levels may be used as a biomarker to correlate with the severity of cognitive impairment in AD patients (Lai et al., 2017). Further, another inflammatory pathway marker transcription factor, nuclear factor kappa B (NF-κB) activity has been shown to be increased in the brains of AD patients (Hong, 2017).

**Figure 1:**
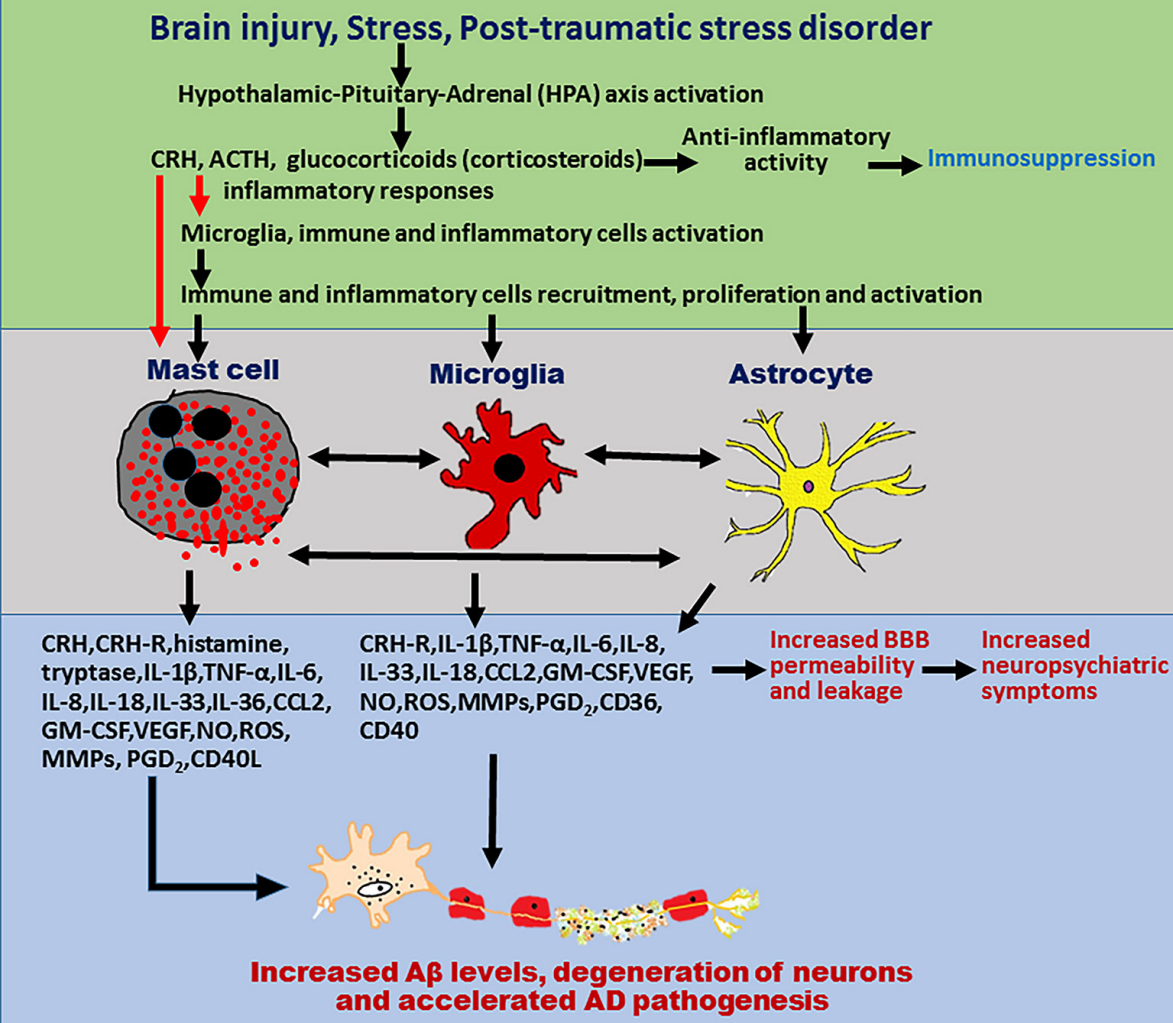
Schematic diagram showing mast cells, brain injury, stress, and PTSD in neurodegeneration in AD pathogenesis. Mast cells-derived inflammatory mediators and direct contact of mast cells with glial cells and neurons, activates brain cells to release additional neuroinflammatory mediators that induce neurodegeneration. Stress conditions activate mast cells through CRH and other neuropeptides to release several neuroinflammatory mediators including cytokines, chemokines, and other neurotoxic mediators such as tryptase, histamine, IL-1β, TNF-α, IL-6, CCL2, IL-8, ROS, CRH, and MMPs. These inflammatory mediators in turn activate glial cells to release additional inflammatory mediators and induce neurodegeneration and neuronal death. Stress also increases BBB permeability and increases the recruitment of immune and inflammatory cells such as mast cell progenitors, mast cells, and T cells in to the brain. In addition, mast cell mediators released from the activated mast cells increase the vascular permeability, increase recruitment of immune, and inflammatory cells to the site of injury and induce neuroinflammatory reactions. These newly recruited mast cell progenitors, mast cells, and T-cells proliferate, mature, and further activate glial cells and neurons by releasing inflammatory and cytotoxic mediators leading to chronic state of neuroinflammation and neurodegeneration. PTSD also induces neuroinflammation and neurodegeneration through immune and inflammatory cells such as mast cells, T-cells, and glial cells in the brain. Mast cells, brain injury, stress conditions, and PTSD comorbidity could induce chronic neuroinflammation and neurodegeneration in neurodegenerative diseases including AD.

Mast cells are present adjacent to the neurons and glial cells in thalamus, hypothalamus, and leptomeninges and activate them by cell-to-cell contacts as well as by releasing inflammatory and neurotoxic mediators (Theoharides et al., 2013). Mast cells located at the brain side (>95%) of the BBB protect the brain from invading pathogens and toxic substances from the peripheral organs (Dong et al., 2014b). Mast cells play a major role in neuroinflammatory conditions including neurodegenerative diseases, stroke, MS, TBI by increasing the BBB permeability and activating the brain resident immune cells microglia, and T-cells (Nakae et al., 2006; Ribatti, 2015). Mast cells are implicated in the brain injuries, stress, and PTSD-induced neuroinflammation that could contribute to the pathogenesis of AD as shown in Figure 1. About 50% of histamine in the brain is released from the brain mast cells (Chikahisa et al., 2013). Mast cells activate glial cells and neurons through protease-activated receptor-2 (PAR-2) pathway (Yuan et al., 2010). These findings strongly suggest that mast cells are important mediators of neuroinflammation. Activation of glia is implicated in neuroinflammation-mediated neurodegeneration mechanisms (Colombo and Farina, 2016). Several studies have shown that targeting glial cells-mediated neuroinflammation is effective in the treatment of AD (McGeer and McGeer, 2015; Bolos et al., 2017; Spangenberg and Green, 2017). As mast cells are implicated in brain injury, stress, and PTSD, activation of mast cells in these conditions could increase neuroinflammation and thereby accelerate the onset and progression of AD. Therefore, the focus of this article is to review currently available data to link the mast cell activation in stress, brain injury, and PSTD with that of pathogenesis of AD. We have searched PubMed for studies in this research area using the keywords mast cells, stress, brain injury, PTSD, and AD.

## Brain injury, mast cells, and inflammation

Neuroinflammation plays central role in the CNS disorders such as MS, PD, AD, brain and spinal cord injuries, stroke, depression, schizophrenia, and chronic pain (Mcgeer and Mcgeer, 2010; Anisman and Hayley, 2012; Skaper et al., 2014; Domingues et al., 2017; Mcmanus and Heneka, 2017; Sawikr et al., 2017; Schain and Kreisl, 2017). Increased levels of proinflammatory cytokines and chemokines also induce behavioral and pathological changes in the brain disorders such as AD and stroke (Rothwell and Strijbos, 1995; Anisman and Merali, 2003; Dantzer and Kelley, 2007). TBI is an important cause of morbidity and mortality in veterans. The initial neuroinflammatory responses after the primary brain injury is beneficial but lingering and chronic immune and neuroinflammatory responses cause additional secondary brain damage (Vink et al., 2003; Skaper et al., 2014; Corrigan et al., 2016). TBI activates glial cells, neurons and immune cells, induces neuroinflammation and neurodegeneration, axonal degeneration, elevates brain and peripheral inflammatory cytokines and chemokines, increases triggering receptor expressed on myeloid cells 2 (TREM2) expression and affects microvascular system (Elder, 2015; Elder et al., 2015). However, studies also suggest suppression of AD symptoms after TBI. Activation of immune cells and changes in the microvascular unit where mast cells are located upregulates the neuroinflammatory responses. Brain injury causes cognitive impairment, increases the accumulation of amyloid precursor protein (APP), extracellular beta amyloid (Aβ) peptide and intracellular neurofibrillary tangles (NFTs) consisting of tau protein associated with inflammatory cytokine release (Johnson et al., 2012; Gupta and Sen, 2016; Kokiko-Cochran et al., 2016; Young et al., 2016). TBI is an important risk factor for neurodegenerative diseases such as dementia and AD (Barnes et al., 2014; Dams-O'connor et al., 2016; Shishido et al., 2016; Collins-Praino and Corrigan, 2017; Mendez, 2017).

Substance P released in TBI or under stress, activates mast cells, microglia and astrocytes, and releases additional neuroinflammatory mediators that increase BBB permeability (Corrigan et al., 2016). There is no specific and approved drug to treat TBI (Tweedie et al., 2016). One study reported that TBI with loss of consciousness influences Lewy body formation, pathogenesis of PD but does not result in dementia and AD (Crane et al., 2016). The first response after TBI is the infiltration and degranulation of mast cells in the brain (Moretti et al., 2016; Hendriksen et al., 2017). Mast cell degranulation releases cytoplasmic granule's prestored histamine and proteases. Mast cells increase BBB permeability and allow inflammatory cell infiltrations in the brain after cerebral ischemic brain injury (McKittrick et al., 2015). Headache associated with mild TBI is due to persistent dural mast cell degranulation and its inflammatory mediator release (Levy et al., 2016). Inhibition of mast cell activation could be used as an initial adjuvant therapy to treat hypoxia-ischemia, ischemic stroke, and ICH in new-borns and adults (Strbian et al., 2009). Increased mast cell numbers and their degranulation-derived histamine mediates ischemia-induced neuronal death in the brain (Biran et al., 2008). Mast cell number increases for days and weeks and contributes to the brain damage by releasing inflammatory mediators such as TNF and IL-9 in perinatal hypoxic-ischemia and that inhibition of mast cell activation decreased the brain damage in the immature rat brains (Jin et al., 2007; Ziemka-Nalecz et al., 2017). Similarly, inhibition of mast cell activation inhibits hemorrhage formation in thrombolytic ischemic stroke in rats (Strbian et al., 2007). Further, TBI has been shown to increase the number of mast cells in the injured cortical area (Lozada et al., 2005). A recent study reported that mast cells are the first responders in ICH and promote BBB breach, edema formation, recruit inflammatory cells, and amplify brain injury (Yehya and Torbey, 2017). These findings clearly indicate that mast cells play an important role in the neuroinflammatory responses after the brain injuries or TBI and that the enhanced neuroinflammation can predispose to the pathogenesis of AD.

## Stress, mast cells, and inflammation

Several inflammatory conditions are worsened by stress, and mast cell activation with inflammatory mediator release plays a crucial role in stress dependent inflammatory mechanism (Theoharides et al., 2004). A recent study showed chronic mild stress for 3 weeks increased the number of mast cells in the brain and disturbed sleep in mice (Chikahisa et al., 2017). Interaction between hypothalamus, pituitary, and adrenal gland is an important stress response in neuropsychiatric conditions and depression (Anisman and Merali, 2003). Stress activates hypothalamo-pituitary-adrenal (HPA) axis within seconds and increases the release of CRH and arginine vasopressin (AVP) from the paraventricular nucleus (PVN) of the hypothalamus in the brain, enhances mast cell activation, vascular permeability, and the expression of CRH receptors (Lytinas et al., 2003; Claes, 2004; Theoharides et al., 2004). Proinflammatory cytokines also activates HPA axis (Hayley, 2011). Furthermore, stress causes CRH release from hypothalamus into the pituitary gland and releases adrenocorticotropic hormone (ACTH) from the pituitary gland. ACTH acts on adrenal cortex to release glucocorticoids, which in turn inhibit the release of CRH and ACTH in a negative feedback mechanism. CRH and ACTH directly activate microglia to release neuroinflammatory mediators (Karagkouni et al., 2013). Psychological and environmental stress conditions induces the release of CRH. CRH also called as corticotropin-releasing factor (CRF), is a 41 amino acid peptide released from hypothalamic neurons as well as from the activated mast cells (Kato et al., 2013). VEGF, an angiogenic cytokine that plays an important role in inflammation, is also elevated in PTSD patients (De Kloet et al., 2008; Pervanidou and Chrousos, 2012). We have shown that stress, CRH, mast cell activation, and VEGF play a crucial role in stress-induced exacerbation of inflammation (Cao et al., 2006a). Further, peripheral derived CRH also augments stress mediated effects (Nozu et al., 2017). We have previously shown that mast cell activation and CRH release under stress conditions increases BBB permeability, and tumor metastases into the brain (Esposito et al., 2001, 2003; Theoharides et al., 2008; Rozniecki et al., 2010). Further, stress induced vascular permeability, an important event in inflammation is reduced in mast cell deficient mice (Kandere-Grzybowska et al., 2003a). Acute stress is reported to accelerate the pathogenesis of neuroinflammatory and autoimmune disease EAE in mice (Chandler et al., 2002).

CRH activates mast cells to release various neuroinflammatory and neurotoxic mediators that leads to a breach of BBB and activates glial cells to release more inflammatory mediators, thereby contributing to the chronic neuroinflammation in the brain (Cao et al., 2006b; Theoharides et al., 2013). Acute stress increases BBB permeability through brain mast cell activation and CRH release (Theoharides and Konstantinidou, 2007). Microglia express CRH receptors, and activation of microglia by CRH from brain cells cause the release of neurotoxic inflammatory mediators in mental disorders (Kritas et al., 2014a). Activation of rat microglia by CRH through CRHR1-induces microglial proliferation and release of TNF-α and activation of mitogen-activated protein kinase (MAPKs) (Kato et al., 2013). CRH also increases microglial expression of IL-18 which is implicated in stress, depression, and PTSD conditions (Kato et al., 2013). A recent report using mast cell and CRHR1 deficient mice reported that CRHR1 mediates stress-induced mast cell degranulation (Ayyadurai et al., 2017). Inflammatory mediators released from microglia induce neuronal destruction in neurodegenerative diseases (Kritas et al., 2014a). Several stressors increase aging-like process and activate microglia toward proinflammatory phenotype causing destruction of neurons (Brown and Vilalta, 2015; Calcia et al., 2016; Niraula et al., 2016). Stress conditions clearly activate microglia in the brain (Delpech et al., 2015). Microglial activation plays an important role in the pathogenesis of neurodegenerative diseases (Perry et al., 2010; Perry and Holmes, 2014; Perry, 2016). Microglia and mast cells which develop from hematopoietic progenitors are reported as two tracks to the road to neuroinflammation (Skaper et al., 2012).

We have previously shown that human mast cells synthesize and secrete CRH and express functional CRH receptors (CRH-R1 and CRH-R2) (Kempuraj et al., 2004; Cao et al., 2005; Papadopoulou et al., 2005). CRH released from mast cell acts in an autocrine as well as paracrine manner to activate mast cells and glial cells in the CNS in stress and neuroinflammatory conditions (Karagkouni et al., 2013; Kritas et al., 2014b). Social stressful experience is associated with psychiatric disorders with enhanced inflammatory responses (Finnell and Wood, 2016). The level of stress-related pathology varies from one person to another, due to the differences in the levels of immune and inflammatory response specifically mast cell response to various stressors and activation signals (Forsythe and Ennis, 2000; Finnell and Wood, 2016; Gaudenzio et al., 2016). Patients with depression exhibit elevated proinflammatory cytokine levels in the plasma and cerebrospinal fluid (CSF) (Finnell and Wood, 2016). Microglial activation is increased in the brains of depressed patients and this increase is correlated with the severity of the depression (Setiawan et al., 2015). Social stress and depression can affect anyone at any age, gender, ethnicity, and socio-economic background (Finnell and Wood, 2016). Previous reports suggest that stress, depression and PSTD are more prevalent in female than male patients (Breslau, 2002; Finnell and Wood, 2016; Mendoza et al., 2016). Psychological and environmental stress induces or worsens anxiety and depression, activates neurons, microglia, and induces neuronal dystrophy (Wohleb, 2016; Mondelli et al., 2017). Stress due to cold worsens neuroinflammation, induces oxidative stress, neuronal autophagy, and enhances immune responses (Qu et al., 2017; Theoharides, 2017). Mast cells are linked with inflammatory pathways leading to chronic depression in mastocytosis patients (Georgin-Lavialle et al., 2016). Mastocytosis patients also show increased oxidative stress markers of inflammation (Gangemi et al., 2015). Stress worsens several conditions such as migraines, by activating mast cells to release inflammatory mediators (Theoharides et al., 1995; Castellani et al., 2010). Stress is undoubtedly linked to the severity of neurodegenerative disorders. Work-related stress such as job security, job environment and lack of job satisfaction increases the risk for vascular dementia and AD (Andel et al., 2012). CRH released in stressful conditions is protective at a lower concentration, but is harmful at higher concentrations and exacerbates AD progression (Pardon, 2011).

Chronic stress can accelerate AD pathogenesis in human and animal models through increases in inflammatory responses, Aβ accumulation, tau hyperphosphorylation, oxidative stress, mitochondrial impairment, and glucose metabolism (Machado et al., 2014). Stress in early-life increases the risk of cognitive disorders in the aged mouse model of AD (Hoeijmakers et al., 2017). Early life chronic stress in transgenic APP/PS1 AD mice from postnatal day 2 to 9 has been shown to increase Aβ pathology, neuroinflammatory mediators, and neuroinflammatory responses in 4 and 10 months-old mice as compared to age matched-wild type mice. Additionally, early life stress induced both immediate as well as late effects; increased inflammatory responses, CD68 expression and neuroinflammatory levels in the hippocampus in an age-dependent manner in AD mouse model (Hoeijmakers et al., 2017). Adolescent stage stress exposure affect brain development, causes depression and brain dysfunctions in the adulthood (Eiland and Romeo, 2013). Chronic stress conditions create a vicious cycle of increased microglial dysfunction associated decreased clearance, and increased Aβ accumulation exacerbating neuroinflammation and neurodegeneration. The mechanism that chronic stress contributes to microglia-mediated neuroinflammation and cognitive impairments in AD is not yet clearly known and that therapeutic interventions to stress-mediated effects could delay the onset, progression, and severity of AD (Piirainen et al., 2017). CD33, CD36, and TREM2 are implicated in stress and AD progression and that stressful conditions exacerbate Aβ pathology in the animal models of AD (Piirainen et al., 2017). Increased cortisol levels and HPA axis dysregulation have been implicated in stress conditions and AD. Chronic stress has been shown to release more Aβ, trigger and worsen AD severity (Morgese et al., 2017). A recent study reported that Tg-AD mouse model treated with CRHR1 antagonist R121919 showed decreased stress mediated oxidative damage, prevented the onset of cognitive impairment and dendritic loss and reduced Aβ deposition in the brain (Zhang and Rissman, 2017). They report that stress hormones activate neuronal oxidative stress, which increases the release of additional stress hormones and cause neuronal damage in the hippocampus in AD brains. The authors further suggest that suppression of stress pathways might be an effective therapy for AD (Zhang and Rissman, 2017).

Chronic stress and short-term modern life-like stress upregulates AD severity in 3xTg-AD mice, an animal model of AD (Baglietto-Vargas et al., 2015). They report that combined emotional and physical stress lasting for 5 h significantly impaired memory in these AD mice as compared to wild type mice. Further, these stress conditions reduced the number of dendritic spines and increased Aβ levels in these 3xTg-AD mice (Baglietto-Vargas et al., 2015). Neurotensin, a neuropeptide along with CRH augments mast cell activation and release of excessive inflammatory mediators in stressful situations (Alysandratos et al., 2012). Immobilization (restrain) stress induces HPA axis and activates intracranial mast cells to release tryptase, a mast cell specific inflammatory protease within 30 min in rats as shown by light and electron microscopy. Pretreatment of these animals with anti-CRH before stress inhibited intracranial mast cell activation (Theoharides et al., 1995). Chronic psychological stress is a risk factor for dementia and AD by inducing microglial proinflammatory status (Piirainen et al., 2017). These reports strongly indicate that mast cells play a crucial role in stress responses associated with inflammation that may predispose to AD pathogenesis in high-risk groups.

## PTSD, mast cells, and inflammation

PTSD is a traumatic stress-related emotional disorder associated with chronic low-grade inflammation (Zass et al., 2017). PTSD causes behavioral impairments as well as immunological disorders (Banks et al., 1995). PTSD is an important concern in war veterans and the combat soldiers at the war regions, and they are at a high risk of developing this disorder. U.S deployed over 2.5 million service members to Iraq since 2001 and about 15% of them developed PTSD after their combat experience. PTSD also affects civilians following trauma or loss of family members (Dursa et al., 2014; Wang and Young, 2016). PTSD patients show chronic stress responses along with low-grade inflammatory reactions in the body (Zass et al., 2017). Most of the people with trauma did not develop PTSD, but only some people develop PTSD. This is because of the other factors such as stress and socio economic factors that may be considered as important risk factors for the pathogenesis of PTSD (Digangi et al., 2013). However, the exact mechanism of the pathogenesis of PTSD is not yet clearly known and therefore no effective treatment options are currently available. Recent studies have shown that immune disorders with excessive inflammatory reactions are present in PTSD patients. Additionally, mast cells, the soldiers of our body's (innate and acquired) defense system are also dysregulated in the combat soldiers. Therefore, it is necessary to urgently understand the pathogenesis of PTSD to take care of our affected defense personnel and to prevent the pathogenesis of PTSD in general. Several reports strongly suggest elevated neuroinflammation in depressed patients and in chronic stress conditions (Zass et al., 2017). Also, behavioral disorders and neuroinflammation are closely linked with peripheral inflammation in social stress model of PTSD (Muhie et al., 2017).

It has been suggested that PTSD, depression, and stress induces low-grade chronic inflammation that could lead to neurodegenerative diseases. Chronic stress, depression, and PTSD in war zone soldiers may be responsible for the increased inflammatory reactions in the active duty American soldiers (Groer et al., 2015). Mast cells could play an important role in the battlefield soldiers due to stress, mood, fearful behavioral activities, and change in the outer environmental conditions. Mast cell numbers are high in the skin, gastrointestinal tract, and respiratory tract through which the body is directly exposed to outer environment. Changes in the outer environmental conditions such as outside temperature (cold or hot), light and darkness, odors, and toxic substances in the air affects/activates mast cells in the barrier regions and increases their numbers to respond (Nakamura et al., 2017). For example, outer environmental cold and low humidity conditions activates skin mast cells, allergen/toxins in the air activates nasal, airways, and ocular regions mast cells and causes acute or chronic allergic and inflammatory reactions (Petra et al., 2014). Additionally, human behavioral and emotional conditions also influences mast cell activations and their numbers in specific regions of the body including in the brain areas (Nautiyal et al., 2008).

A previous report suggests that PTSD-like trauma with high CRH level induces dementia and AD pathogenesis (Justice et al., 2015). Studies have linked PTSD with inflammation related diseases, elevated inflammatory responses, and accelerated aging process (Tsai et al., 2011; Wolf and Schnurr, 2016; Lindqvist et al., 2017). Peripheral inflammatory markers and total inflammatory scores are shown to be higher in combat experienced veterans with PTSD, as compared to veterans without PTSD (Lindqvist et al., 2017). It is interesting to note that comorbidity of TBI and PTSD shows augmented inflammation, associated with elevated IL-6 and TNF-α levels, and this increase correlates with the severity of PTSD symptoms (Devoto et al., 2016). Similarly, studies have also shown elevated levels of IL-6, IL-1β, TNF-α, and IFN-γ in PTSD patients (Cohen et al., 2011; Passos et al., 2015). These cytokines can bind to receptors in the glial cells and phosphorylate MAPKs, which leads to the activation of nuclear factor-kappa B (NF-κB) and release of inflammatory mediators. Supporting this notion, we have previously reported that IL-1 activates mast cells to release IL-6 through the activation of p38 MAPK (Kandere-Grzybowska et al., 2003b, 2006; Castellani et al., 2009). A recent report indicates that chronic stress and anxiety increases the rate of PTSD (Zass et al., 2017). Systemic inflammation marker, CRP is increased in depression as well as in PTSD patients, indicating that PTSD patients show enhanced inflammatory responses (Powers et al., 2016; Miller et al., 2017; Solomon et al., 2017). Thus, it has been suggested that certain inflammatory markers can be used as biomarkers for PTSD (Cohen et al., 2011).

A study conducted on Iraq and Afghanistan deployed US-veterans under the age of 55 that were associated with PSTD and endocrine and immune abnormalities reported increased risk for developing autoimmune diseases such as MS, rheumatoid arthritis, thyroiditis, lupus erythematosus, and inflammatory bowel disease (IBD) (O'donovan et al., 2015). Hence, there is a clear evidence that PTSD is an important risk factor for several autoimmune and mast cell associated diseases. Cytokines and chemokines including TNF-α, IL-6, and IL-1β cross BBB and induce neuroinflammation directly, and also by activating inflammatory, glial, and neuronal cells in the brain (Banks et al., 1995). Further, inflammatory cytokines released from activated glial cells, and inflammatory cells contribute to the chronic pain in PSTD (Lerman et al., 2016). Additionally, mast cell-derived inflammatory mediators including SP strongly contribute to the pain in PTSD patients. Stress increases the release of CRH and this CRH activates HPA axis in stress responses in PTSD patients with depression (Mendoza et al., 2016). This elevated CRH increases the vascular permeability and activates glial cells and inflammatory cells such as mast cells to release additional inflammatory mediators (Mendoza et al., 2016). Moreover, mast cells also contribute to the increased level of CRH in the brain, as mast cells are target as well as source for CRH as we reported previously (Kandere-Grzybowska et al., 2003b; Kempuraj et al., 2004). During the stress conditions, elevated inflammatory mediators cross BBB and induce/augment neuroinflammation that accelerates the pathogenesis of AD. These findings indicate that stress accompanied with chronic inflammation in PTSD could lead to neurodegeneration in diseases such as AD. Moreover, PTSD is a known risk factor for dementia in the veterans and also in civilians (Flatt et al., 2017). However, mast cells are clearly involved in peripheral and CNS inflammation and in various stress conditions and trauma, but the exact role of mast cells is not yet studied in PTSD patients. Moreover, mast cells could increase both acute and chronic inflammatory reactions in stress, brain injuries, and PTSD and could contribute to the development and progression of AD (Figure 1).

## Mast cells, neuroinflammation, and AD

AD is the most prevalent chronic progressive neurodegenerative disease associated with dementia, neuroinflammation, and neurodegeneration (McGeer et al., 2016). AD is considered as an inflammatory disease involving immune components in the brain (Heneka et al., 2015; McGeer and McGeer, 2015; Bolos et al., 2017). AD pathogenesis begins even 10 years before the clinical symptoms are identified in AD patients (McGeer et al., 2016). There are no effective drugs to treat neurodegeneration in AD since the disease mechanisms are not yet clearly understood. Anti-inflammatory and anti-depressants can reduce neuroinflammation in dementia and AD (Hashioka et al., 2009; McGeer et al., 2016). Inflammatory mediators induce neuroinflammation, synaptic dysfunction, hyperphosphorylated tau generation, Aβ production, and neurodegeneration in the brain (Mohammadzadeh Honarvar et al., 2017). Anti-inflammatory drugs are neuroprotective and suppress disease progression by reducing Aβ generation and its accumulation, inflammatory mediator release with improvement of cognitive functions (Budni et al., 2016; McGeer et al., 2016; Mohammadzadeh Honarvar et al., 2017). Aβ pathology and normal aging processes are associated with activation of immune as well as inflammatory cells in the brain. Neuroinflammation and neurodegeneration are vicious cyclic processes in neurodegenerative diseases and in normal aging processes. Available current research evidence indicates that neuroinflammation clearly contributes to the neurodegeneration in several neurodegenerative diseases (Glass et al., 2010; Kempuraj et al., 2016b, 2017b). However, the exact mechanism of neuroinflammation is not yet clearly understood. Neuroinflammation is a complex mechanism involving different immune and inflammatory cells and different inflammatory mediators in AD pathogenesis, brain injuries, and stress conditions. The currently available anti-inflammatory drugs are not affecting all or the key inflammatory mediators or genes in these conditions. Moreover, these diseases and disorders are multifactorial and the anti-inflammatory drugs or antidepressants need to affect specific inflammatory pathways and inflammatory mediators in specific diseases for its maximal beneficial effects. BBB is one of the main problems in delivering the drugs that acts on neuroinflammation. These drugs should also be effective in stress-mediated hormonal disorders in addition to neuroinflammation (Galecki et al., 2018). CRH-antagonists could be therapeutic agents in stress-related CNS and peripheral inflammatory disorders (Nezi et al., 2000). More importantly, anti-inflammatory and other currently available drugs do not completely stop the onset or progression of the disease and do not induce regeneration of neurons and its network connections in the affected brain regions. Continued and extensive research in this field is essential to achieve this goal in the future.

Various conditions including stress conditions induce/augment neuroinflammation and AD pathogenesis. Mast cell activation causes either neuroprotection or neuroinflammation based upon the level and number of cells activated (Mukai et al., 2016). Excessive and chronic activation of mast cells lead to neuroinflammation and neurodegeneration. Though mast cells are implicated in neuroinflammatory diseases such as MS, EAE and PD, the role of mast cells in AD pathogenesis is still elusive. Several lines of evidence indicates that mast cell activation could accelerate AD pathogenesis in high-risk group patients with brain injury and trauma, stress conditions, and PTSD. Since mast cells play important role in inflammation, neuroinflammation, stress, and psychiatric disorders, it is important to study its role in the pathogenesis of PTSD-associated neurodegenerative diseases such as AD. Several recent studies show that proinflammatory cytokines and mast cell-derived inflammatory mediators are implicated in neuroinflammation and neurodegeneration in the CNS (Purcell and Atterwill, 1995; Yuan et al., 2010; Banuelos-Cabrera et al., 2014; Dong et al., 2014b; Becher et al., 2017; Spangenberg and Green, 2017). Brain proinflammatory protein glia maturation factor (GMF) which was discovered in our laboratory is involved in the pathogenesis of AD and MS/EAE (Lim et al., 1989; Zaheer et al., 2007, 2011a,b; Stolmeier et al., 2013; Thangavel et al., 2013, 2017). We have recently reported that GMF regulates neuroinflammation through NLRP3 inflammasome in AD brain (Ahmed et al., 2017). Further, we have shown that mast cells also express GMF and suggest that GMF in mast cells may also play an important role in the pathogenesis of neurodegenerative diseases including AD (Kempuraj et al., 2015). Additionally, we have also shown that GMF activates mouse and human mast cells to release mast cell specific proteases and other neuroinflammatory mediators that are implicated in neuroinflammation and neurodegeneration in PD and AD (Kempuraj et al., 2015, 2017a). We have previously reported enhanced expression of IL-33 and GMF at the vicinity of APs and NFTs in human AD brain (Xiong et al., 2014). Further, we demonstrated that incubation of mouse astrocytes with Aβ 1-42 *in vitro* increased the expression of IL-33 indicating IL-33 is implicated in AD (Xiong et al., 2014).

SP is involved in the neurodegenerative diseases. We have shown that IL-33 increases SP-mediated release of inflammatory mediator from mast cells (Theoharides et al., 2010). These results suggest that IL-33 released from astrocytes could activate microglia and mast cells in the brain, as IL-33 is a strong activator of mast cells (Hudson et al., 2008; Castellani et al., 2009; Yasuoka et al., 2011). However, another study showed that injection of IL-33 led to improved memory deficit in APP/PS1 AD mice model (Fu et al., 2016). This suggests that IL-33 could act differently depending upon the environment and concentration. Mast cells are the first immune responding cells in the brain before other cells in certain conditions (Dong et al., 2014b; Hendriksen et al., 2017). Mast cells are suggested as one of the first brain cells that detect and respond early to Aβ formation in the pathogenesis of AD (Harcha et al., 2015; Hendriksen et al., 2017). These studies suggest that mast cells specifically identify the ongoing process in the formation of Aβ in the pathogenesis of AD. The association of mast cells and AD is reported in mastocytosis (increased mast cells in the body) patients. Expression of Aβ peptide, major component of amyloid plaques (APs) in AD and tau-protein has been reported in the skin mast cells of mastocytosis patients (Kvetnoi et al., 2003). Aβ peptide has been reported to activate mast cells to release inflammatory mediators that are implicated in the pathogenesis of AD (Niederhoffer et al., 2009). Increased levels of ROS in AD could activate mast cells to release inflammatory mediators (Chelombitko et al., 2016). Several mast cell-derived inflammatory mediators are reported to be involved in the AD pathogenesis and its level of severity (Shaik-Dasthagirisaheb and Conti, 2016). Mast cells, in fact are similar to neurons with regard to synthesis and secretion of neurotrophic factors, responsiveness to neuropeptides and monoaminergic content such as dopamine (Purcell and Atterwill, 1995).

Mast cells are mostly located in choroid plexus, leptomeninges, and brain parenchyma and form a unit in the neurovascular structure in the CNS (Banuelos-Cabrera et al., 2014). Mast cells migrate and accumulate in the specific region of the brain. Many factors such as cytokines/chemokines, eicosanoids, VEGF, and fibroblast growth factor (FGF), platelet derived endothelial cell growth factor influence the movement, activation and degranulation of mouse mast cells (Gruber et al., 1995). Several neurotrophic factors induce mast cells to release histamine (Purcell et al., 1996) that activates microglia through histamine receptors H1 and H4 to release neurotoxic mediators such as IL-1β, TNF-α, IL-6, and nitric oxide (NO) (Dong et al., 2014a). These proinflammatory mediators directly induce neuronal death in the brain. Inflammatory cytokines such as IL-1β are known to phosphorylate tau (Collins-Praino and Corrigan, 2017; Domingues et al., 2017) and induce neurodegeneration (Rothwell and Luheshi, 2000). Aβ-peptides are normal products of the metabolism that induce localized as well as general inflammatory responses [10]. Elevated intracellular concentration of calcium induces Aβ aggregation in the AD brain and activates human mast cells to release inflammatory mediators (Kempuraj et al., 2005; Theoharides et al., 2010; Koran et al., 2014). The number of mast cells in the normal brain is less; however, the number increases in the affected regions in AD brains (Banuelos-Cabrera et al., 2014).

Mitochondrial uncoupling proteins (UCPs) are implicated in neurodegenerative diseases. We have recently reported downregulation of the expression of UCPs 2 and 4 in the parahippocampal gyrus of AD brains where GMF expression is also increased (Thangavel et al., 2017). We have also previously shown that mast cells express UCP2 and UCP 4 (Tagen et al., 2009; Kempuraj et al., 2016a) and GMF (Kempuraj et al., 2015). Further, we showed that deficiency of UCP2 in mouse mast cells decreased the release of inflammatory mediators such as IL-6, PGD_2_, and histamine with inhibition of MAPKs activation (Tagen et al., 2009). Another study has shown that UCP2 deficient microglia released more NO and IL-6 when stimulated with lipopolysaccharide (De Simone et al., 2015). These findings show that mast cell UCPs could be implicated in the pathogenesis of AD.

Although recent reports suggest that mast cells and CRH are crucial in the pathogenesis of AD, there are some controversies regarding this hypothesis (Rehman, 2002; Pardon, 2011). The concentration of CRH has been reported to be higher in the regions prone to develop AD related pathological changes (Pedersen et al., 2001). Mast cell activation induces postoperative cognitive dysfunction (POCD) after surgical procedure-mediated neuroinflammation (Li et al., 2017). Chymotrypsin-like protease was reported in the meningeal and intracortical perivasculature where there is high Aβ accumulation in rat AD brains. This chymotrypsin-like protease has been suggested to influence aggregation of Aβ deposition (Nelson et al., 1993). Mast cells express IgE receptor (FcεRI), release histamine and play important role in mediating various allergic reactions (Frieri et al., 2015; Arthur and Bradding, 2016). Presence of allergic diseases such as asthma in which mast cells are increased as well as heavily activated are strongly linked to phosphorylation of tau, dementia, and AD (Chen et al., 2014; Bolos et al., 2017; Galli, 2017). We have recently reported that mast cell activation in several inflammatory conditions in the periphery could increase neuroinflammation and neurodegeneration (Kempuraj et al., 2017b). These findings show that mast cells are implicated in the pathogenesis of AD. Moreover, it has been shown that mast cells play important role in stress induced severity of asthma (Theoharides et al., 2012b). Stress conditions exacerbate asthma severity by increasing the number of mast cells as well as increased number of activated mast cells.

## Conclusions and perspectives

Mast cell activation is implicated in neuroinflammation, brain injuries, and various stress conditions. We suggest that mast cells participate in the pathogenesis of AD, and this process could be accelerated and worsened in brain injury, stress, and PTSD comorbidity. Inhibition of mast cell-associated inflammatory pathways in brain injury, stress, and PTSD could be explored as a new therapeutic target to inhibit or prevent the pathogenesis and potentially delay the onset of AD. Though the evidence is currently limited, investigating the role of mast cell activation in brain injuries, stress, and PTSD comorbidity in the onset and progression of AD is an important emerging new area to understand and to effectively treat neuroinflammatory disorders including AD.

## Author contributions

DK: Conceptualization, wrote, and edited the manuscript; AZ: Funding acquisition, provided resources and edited the manuscript; GS, RT, MA, SZ, SR, SI, SB, and SB-R: Edited the manuscript.

### Conflict of interest statement

The authors declare that the research was conducted in the absence of any commercial or financial relationships that could be construed as a potential conflict of interest.
